# Supplementary material for: Comparative multiomics analysis of cell physiological state after culture in a basket bioreactor
Source: Sci Rep. 2022 Nov 23;12:20161. doi: 10.1038/s41598-022-24687-4 (PMC9686226; doi:10.1038/s41598-022-24687-4)
Supplement: Supplementary file 1 — Supplementary Information 1. [file 41598_2022_24687_MOESM1_ESM.zip › raw data/Transcriptomic raw data/7.AS/2.dasplot/TE1vsTC1/A3SS/13_SNED1_NW_023666040.1_72243319_72243694_-@NW_023666040.1_72243319_72243698_-@NW_023666040.1_72244325_72244362_-.pdf]

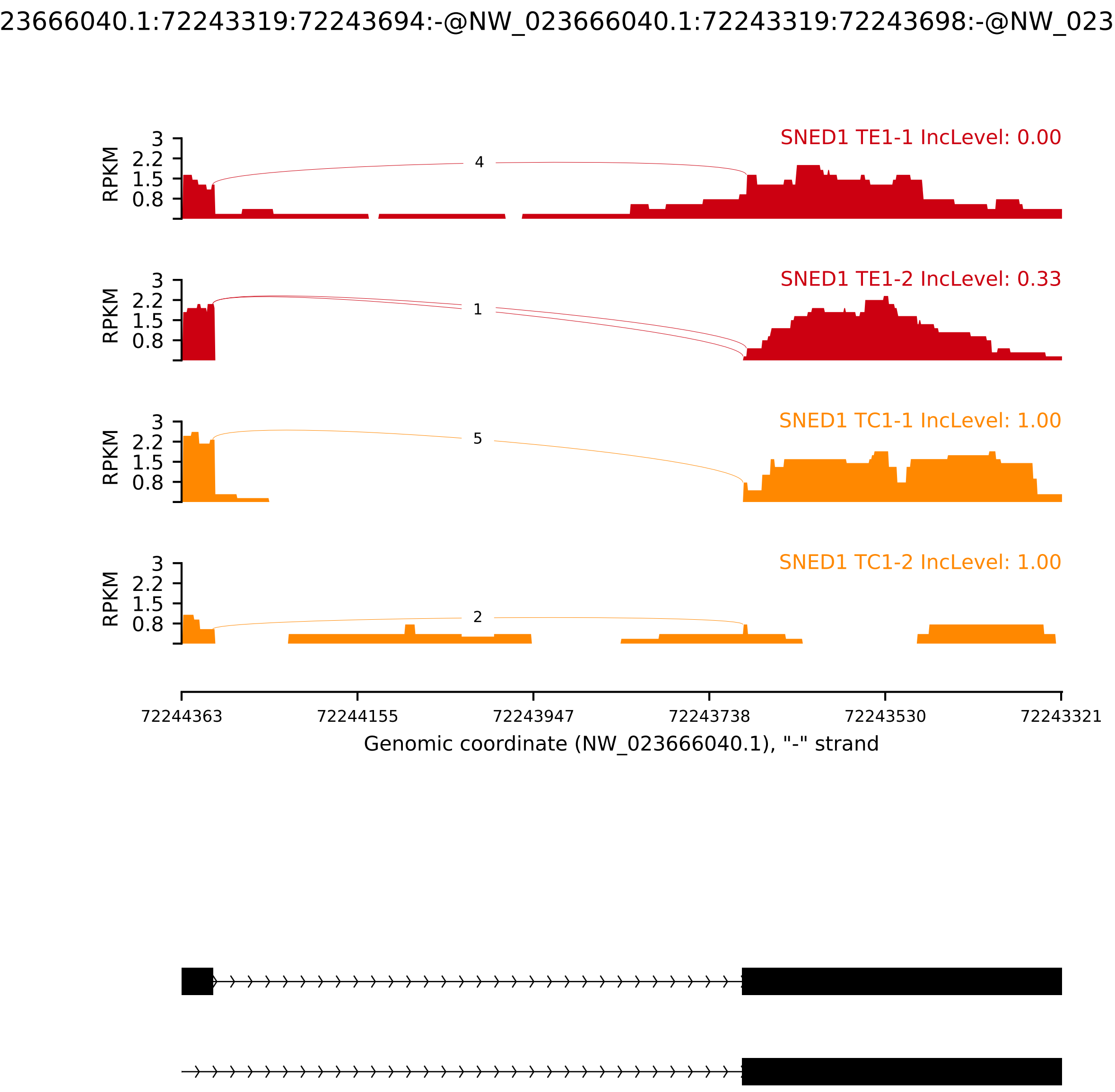
23666040.1:72243319:72243694:-@NW_023666040.1:72243319:72243698:-@NW_023666040.1:72244325:72244
SNED1 TE1-1 IncLevel: 0.00
SNED1 TE1-2 IncLevel: 0.33
SNED1 TC1-1 IncLevel: 1.00
SNED1 TC1-2 IncLevel: 1.00
RPKM
3
2.2
1.5
0.8
4
1
5
2
72244363
72244155
72243947
72243738
72243530
72243321
Genomic coordinate (NW_023666040.1), "-" strand
